# High‐level performances following low altitude training and tapering in warm environments in elite racewalkers

**DOI:** 10.1002/ejsc.12161

**Published:** 2024-07-11

**Authors:** Bastien Krumm, Brent Vallance, Louise Burke, Johan Garcia, Janne Bouten, Franck Brocherie, Jonas J. Saugy, Francesco Botrè, Raphael Faiss

**Affiliations:** ^1^ Research and Expertise in Anti‐Doping Sciences—REDs Institute of Sport Sciences University of Lausanne Lausanne Switzerland; ^2^ Exercise and Nutrition Research Program Mary Mackillop Institute for Health Research Australian Catholic University Melbourne Victoria Australia; ^3^ Athletics Australia Melbourne Victoria Australia; ^4^ Laboratory Sport Expertise and Performance (EA 7370) French Institute of Sport (INSEP) Paris France

**Keywords:** athletes, endurance, environmental physiology, physiology, racewalking

## Abstract

Current guidelines for prolonged altitude exposure suggest altitude levels ranging from 2000 to 2500 m to optimize an increase in total hemoglobin mass (Hbmass). However, natural low altitude locations (<2000 m) remain popular, highlighting the interest to investigate any possible benefit of low altitude camps for endurance athletes. Ten elite racewalkers (4 women and 6 men) underwent a 4‐week “live high‐train high” (LHTH) camp at an altitude of 1720 m (P_I_O_2_ = 121 mmHg; 20.1°C; 67% relative humidity [RH]), followed by a 3‐week tapering phase (20 m; P_I_O_2_ = 150 mmHg; 28.3°C; 53% RH) in preparation for the World Athletics Championships (WC). Venous blood samples were withdrawn weekly during the entire observation period. In addition, blood volumes were determined weekly by carbon monoxide rebreathing during altitude exposure and 2 weeks after return to sea level. High‐level performances were achieved at the WC (five placings among the Top 10 WC races and three all‐time career personal bests). A slight but significant increase in absolute (+1.7%, *p* = 0.03) and relative Hbmass (+2.3%, *p* = 0.02) was observed after 4‐week LHTH. In addition, as usually observed during LHTH protocols, weekly training distance (+28%, *p* = 0.02) and duration (+30%, *p* = 0.04) significantly increased during altitude compared to the pre‐LHTH period. Therefore, although direct causation cannot be inferred, these results suggest that the combination of increased training load at low altitudes with a subsequent tapering period in a warm environment is a suitable competition‐preparation strategy for elite endurance athletes.

## Highlights

- Despite current guidelines for prolonged altitude exposure, natural low altitude locations (<2000 m) remain popular among elite endurance athletes, highlighting the interest to investigate any possible benefit of low altitude camps.
- This study reports that elite racewalkers achieved a modest but significant increase in Hbmass after a 4‐week LHTH camp at an altitude of 1720 m. Furthermore, this intervention led to performance success at the subsequent World Athletics Championships.
- The combination of an increased training load while exposed to low altitude is suggested as a valuable competition preparation strategy for elite endurance athletes.

## 1 INTRODUCTION

To further increase their training stimuli, elite endurance athletes engage in altitude training, with the pioneering models involving “live high‐train high” (LHTH) camps (Adams et al., 1975; Faulkner et al., 1967). By benefiting from altitude for its accelerating effect on erythropoiesis, the main objective of such a training protocol is to provide improved oxygen convective transport through an increase in the total hemoglobin mass (Hbmass) (Levine & Stray‐Gundersen, 1997; Mairbäurl et al., 1986). Hence, with a strong correlation between Hbmass and maximal oxygen uptake (Saunders et al., 2013), an improvement in performance upon return to sea level has been reported in such context (Hauser et al., 2016). “Live high‐train low” (LHTL) approaches subsequently evolved, with the primary aim of avoiding the hypoxia‐associated compromise in training intensities (Levine & Stray‐Gundersen, 1997; Sharma, 2022). Although non‐hematological mechanisms including improved muscle efficiency (e.g., greater buffer capacity) might contribute to sea‐level performance improvements (Gore et al., 2007), the main benefits following prolonged hypoxic exposure seem related to the erythropoietic pathways (Levine & Stray‐Gundersen, 2005). Indeed, current recommendations advise a 3 to 4‐week exposure, ≥12–16 h a day, to altitudes of between 2000 and 2500 m to achieve an increased Hbmass, while altitudes below 2000 m are generally considered insufficient to provide a measurable erythropoietic stimulation (Chapman et al., 2014; Wilber, 2007).

Considering the total duration of exposure and the severity of environmental hypoxia as key determinants of hematological adaptations, it is however proposed that a similar hypoxic dose could be reached by continuous lengthier exposure to lower altitudes (Garvican‐Lewis et al., 2016). In addition, notwithstanding these established guidelines and the increasing assimilation of sports science within the athlete's journey (Chamari, 2019), many athletes have persisted in implementing LHTH approaches at lower altitudes (e.g., from 1300 to 2000 m as defined by Bärtsch and Saltin (2008)) as part of their preparation for major events (Sharma, 2022; Turner et al., 2019). For example, there are multiple case studies of such low hypoxic dose‐based strategies involving elite athletes with highly favorable outcomes (Frese & Friedmann‐Bette, 2010; Garvican‐Lewis et al., 2015; Sharma et al., 2019).

Although the potential benefits of altitude training for sea‐level performance have been extensively investigated, controlled studies including elite endurance athletes completing altitude training camps under ecologically valid conditions are scarce (Sharma, 2022). Moreover, the wide intra‐ and inter‐individual variability regularly observed in hematological response to prolonged hypoxic exposure encourages further investigation (Hauser et al., 2017; Nummela et al., 2021). This study investigated the hematological responses and performance outcomes in elite racewalkers during and after the LHTH camp at low natural altitudes (1720 m) preceding a major international event (2023 World Athletics Championships [WC]). Since low altitude training camps may result in positive outcomes for individuals on their return to sea level, we hypothesized that elite endurance athletes would achieve performance improvements.

## 2 METHODS

### 2.1 Participants

Ten elite racewalkers, including four women and six men, were monitored for 7 weeks. All participants can be categorized from elite/international to world‐class level (McKay et al., 2022). Out of the 10 athletes, 8 had prior experience with altitude training. All athletes lived between sea level and 500 m and had not been exposed to hypoxia during the 2 months leading up to the current protocol. Their anthropometric characteristics at the beginning of the study are summarized in Table 1. All participants were informed of the procedures and risks involved in taking part in the study. Despite the absence of any prior anti‐doping rule violation in the participants, the use of prohibited substances or doping methods cannot be ruled out completely. The participants were however informed that they could be subject to regular anti‐doping controls during the study, and we therefore assumed that doping could not be considered as a significant confounder in any physiological change observed during the study.

**TABLE 1:** Anthropometric characteristics and hematological data of the study population at the beginning of the study.

	Men (*n* = 6)	Women (*n* = 4)
Anthropometric characteristics		
Age (yrs)	30.3 ± 5.4	22.0 ± 2.8
Mass (kg)	68.0 ± 4.4	58.0 ± 5.1
VO_2max_ (mL⋅min⋅kg^−1^)	70.6 ± 3.7	61.4 ± 1.9
Training duration (h⋅week^−1^)	9.0 ± 0.9	7.8 ± 1.1
Training distance (km⋅week^−1^)	94.7 ± 12.6	96.7 ± 7.9
Hematological data		
Hematocrit (%)	47.4 ± 1.9	40.1 ± 2
Hemoglobin concentration (g⋅dL^−1^)	16.2 ± 0.8	13.1 ± 0.7
Total hemoglobin mass (g)	1003 ± 121	688 ± 92
Relative total hemoglobin mass (g⋅kg^−1^)	14.7 ± 1.8	11.9 ± 0.9
Red blood cell volume (L)	2841 ± 385	2024 ± 253
Plasma volume (L)	3248 ± 438	2952 ± 409
Blood volume (L)	6089 ± 704	4976 ± 658

*Note*: Values reported as means ± SD.

### 2.2 Experimental design

**FIGURE 1:**
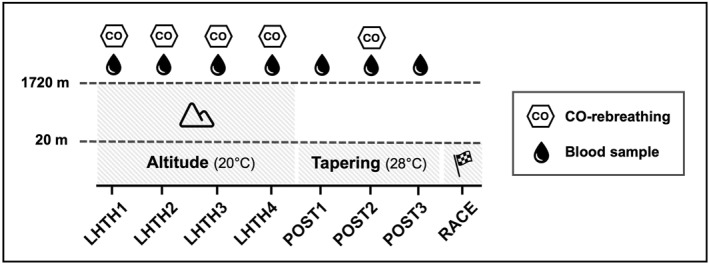
Illustration of the study design.

The athletes spend ∼4 weeks (27 ± 2 days) at an altitude of 1720 m (inspired oxygen pressure [P_I_O_2_] = 121 ± 0.9 mmHg; ambient temperature: 20.0 ± 3.4°C; relative humidity [RH] = 67.4 ± 6.2%; Celerina, Switzerland), before returning to near sea level (20 m; P_I_O_2_ = 150 ± 0.6 mmHg; 28.3 ± 3.3°C; 53.3 ± 10.2% RH; Montpellier, France) for 3 weeks until their competition (100 m; P_I_O_2_ = 148 ± 0.2 mmHg; 30.1 ± 1.8°C; 43.8 ± 3.8% RH; Budapest, Hungary) (Figure 1). The athletes were in a “pre‐competition” phase leading to the WC, which featured as their main competition of the year (20‐km or 35‐km race walk). The researchers did not provide recommendations or direct intervention in the training activities undertaken by these athletes, allowing them to follow the individual guidance of their coach.

### 2.3 Blood sampling

Blood samples were collected on a weekly basis throughout the entire protocol (7 measurements in total), that is, during the LHTH camp (LHTH1–LHTH4) and the tapering period leading to competition (POST1–POST3). The first sample was taken between 24 and 72 h after reaching altitude (LHTH1) and the last sample was collected 48 h before the race. Venous blood samples were drawn by trained phlebotomists from an antecubital forearm vein in 2 mL EDTA tubes (Vacutainer® tubes, Becton Dickinson), with strict adherence to the World Anti‐Doping Agency (WADA) ABP Operating Guidelines (WADA, 2023). Specifically, all blood samples were collected in the morning before the first training session of the day (between 7 and 9 a.m.), and participants rested in a seated position for 10 min prior to sampling. After withdrawal, blood samples were immediately packaged under refrigerated conditions (at 4°C, controlled with a temperature data logger [LogTag^®^]) and sent to the laboratory for analysis within 24 h. Red blood cell count parameters were measured in duplicate by flow cytometry (XN‐1000, Sysmex Corporation), for red blood cells (RBC), hemoglobin concentration ([Hb]), hematocrit (Hct), reticulocyte percentage (Ret%), reticulocyte count (Ret#), and immature reticulocyte fraction (IRF).

### 2.4 Circulating blood volume

Circulating blood volumes were determined weekly during prolonged hypoxic exposure (LHTH1‐4) and 10 days after altitude exposure (POST2) (five measurements in total) by the optimized carbon monoxide (CO)‐rebreathing method using a fully automated instrument (OpCo: Detalo Instruments). The detailed procedure is described elsewhere (Siebenmann et al., 2017). In summary, participants underwent a 6‐min inhalation of a gas mixture containing medical‐grade oxygen (O_2_) and pure CO (99.997%), followed by 4 min of breathing ambient air for gas mixing. Administered CO doses were 1.0 mL kg^−1^ in men and 0.8 mL kg^−1^ in women. Capillary blood samples were taken before and after the 10‐min protocol, with carboxyhemoglobin (HbCO) analyzed in triplicate with a dedicated blood gas analyzer (ABL‐80, Radiometer). The remaining CO in the system was measured by a pre‐calibrated CO sensor (Monoxor Plus, Bacharach) to determine the eventual dose not inhaled. Finally, based on ΔHbCO, [Hb] and Hct, Hbmass, total blood volume (BV), red blood cell volume (RBCV), and plasma volume (PV) were determined with formulas described elsewhere (Siebenmann et al., 2017). The procedure was repeated, and if the difference between these measures exceeded 3%, a third measurement was undertaken. Averaged values from the two closest measurements were then recorded. Using this protocol, the typical error of measurement was 1.6%. Hence, triplicate CO‐rebreathing measurements were performed in 3.9% of all measurement sessions. After the first CO rebreathing test, HbCO values were 6.3 ± 1.1%, after the second test, HbCO increased to 10.4 ± 1.3%, and after the third test to 14.8 ± 0.4%.

### 2.5 Training monitoring

Training content (intensity, duration, and frequency) was documented, with each participant reporting their training sessions (both the total distance covered and the duration) using their personal GPS watch and the rating of perceived exertion (RPE, 1–10 scale) for each walking/running training session (prescribed by their usual national team coach). For training load comparison, weekly averages were calculated before (PRE), during (LHTH1‐4), and after altitude (POST). Training load was calculated by multiplying weekly distance and duration. Additionally, resting heart rate (HR) and peripheral capillary oxygen saturation (SpO_2_) were measured every morning at altitude (at wake‐up after 5 min seated rest) with a pulse oximeter (BPX800, Braun Healthcare). Similarly, sleep quality perception was reported using a visual analog scale (1–10). Finally, local meteorological conditions (ambient temperature, RH, and barometric pressure) were reported daily (data retrieved from https://www.meteosuisse.admin.ch and https://www.accuweather.com).

### 2.6 Data and statistical analyses

Data are presented as means and standard deviations (±SD). Distribution normality was assessed by the D'Agostino & Pearson test. Visual inspection of residual plots allowed the exclusion of any obvious deviations from homoscedasticity. Mixed‐effects analyses were run for each parameter to determine whether changes in the dependent (physiological variables) variables differed over time (fixed factor). A Geisser–Greenhouse correction was applied to prevent sphericity violation. Inter‐individual variation in circulating blood volumes after exposure to altitude was determined by calculating the coefficients of variation (CV, %) based on the differences between LHTH1 and LHTH4 for each participant. 95% confidence intervals were reported for each multiple comparison. All statistical analyses and graphs were carried out using GraphPad Prism 9 software (https://www.graphpad.com). The null hypothesis was rejected for *p* < 0.05.

## 3 RESULTS

Eight of the 10 athletes who participated in the study competed at the WC. Six athletes competed in one race (20‐km or 35‐km Race Walk), while two athletes took part in both events. Overall, athletes within the cohort achieved five placings among the Top 10 of the various races. The average times for the 20‐km race were 01:33:17 and 01:19:57 for women and men, respectively. Similarly, for the 35‐km race, average times were 02:57:55 and 02:26:15. In addition, five annual seasonal bests and three all‐time career personal bests were achieved during the targeted races.

**FIGURE 2:**
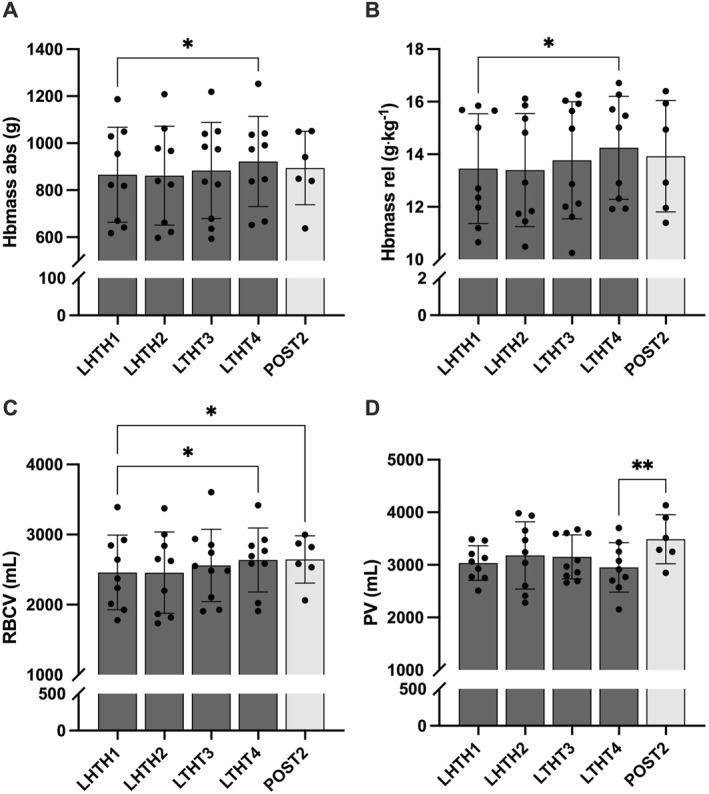
Blood volumes during altitude training (LHTH1‐4) and after 2 weeks back to sea level (POST2). (A–B) Total hemoglobin mass (Hbmass) in absolute (abs) and relative (rel) units, (C) RBCV, and (D) PV. *p < 0.05 and **p < 0.01 for the difference between timepoints. LHTH, live high‐train high; PV, plasma volume; RBCV, red blood cell volume.

**FIGURE 3:**
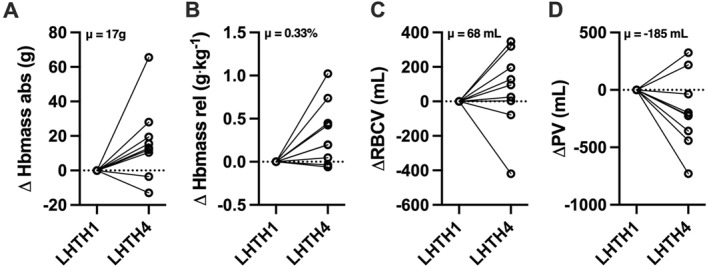
Individual differences in blood volumes between the first (LHTH1) and the last (LHTH4) week of the altitude training camp. (A–B) Total hemoglobin mass (Hbmass) in absolute (abs) and relative (rel) units, (C) RBCV, and (D) PV. LHTH, live high‐train high; PV, plasma volume; RBCV, red blood cell volume.

**FIGURE 4:**
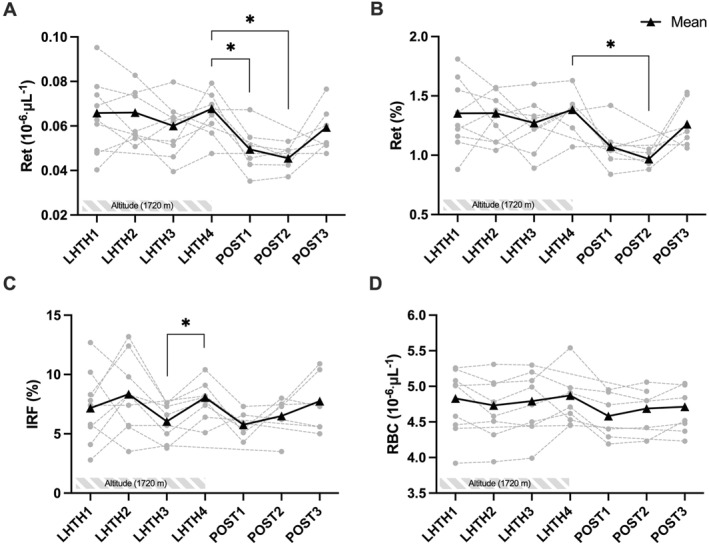
Hematological variables during altitude training (LHTH1‐4) and during the tapering period leading to the competition (POST1‐3). (A) Reticulocytes count (Ret#), (B) Reticulocytes percentage (Ret%), (C) Immature reticulocyte fraction (IRF), and (D) Red blood cells (RBC). *p < 0.05 for the difference between timepoints. IRF, immature reticulocyte fraction; LHTH, live high‐train high; RBC, red blood cell.

Compared to LHTH1 (24–72 h after arrival at altitude), a significant increase in absolute Hbmass (+1.7 ± 1.9%, *p* = 0.03 [95% CI 3.66, −0.27]), relative Hbmass (+2.3 ± 2.7%, *p* = 0.01 [95% CI 4.99, −0.28]), and RBCV (+3.6 ± 8.9%, *p* = 0.03 [95% CI 12.4, −5.16]) was observed at LHTH4 with only RBCV remaining elevated at POST2 (+8.1 ± 6.6%, *p* = 0.04 [95% CI 16.5, −0.41]) (Figures 2 and 3). Out of 10 athletes, an increase in Hbmass was observed in 8, while a decrease was reported in 2. Compared to LHTH4, Ret% (−30.8 ± 7%, *p* = 0.03 [95% CI 7.11, −75.2]) and Ret# (−34.9 ± 5%, *p* = 0.01 [95% CI 18.4, −64.9]) were significantly downregulated at POST2 but not for IRF (−1.3 ± 16%, *p* = 0.58) (Figure 4). Conversely, PV was increased at POST2 (+23.3 ± 7%, *p* = 0.01 [95% CI 41.3, 8.29]). No significant variations were observed for BV, RBC, [Hb], and Hct during the protocol period. Additionally, CV% for absolute Hbmass, relative Hbmass, RBCV, and PV were 134%, 110%, 336%, and 175%, respectively.

Compared to the 4‐week period that preceded the LHTH camp, athletes reported a significant increase in the weekly training distance (+28 ± 24%, *p* = 0.02 [95% CI 48.9, 8.56]), training duration (+35 ± 30%, *p* = 0.04 [95% CI 65.8, 3.79]), and training load (+71 ± 51%, *p* = 0.03 [95% CI 125, 17.3]) during the prolonged hypoxic exposure. Morning HR decreased from LHTH1 (53 ± 8 bpm) to LHTH4 (48 ± 6 bpm, *p* = 0.01 [95% CI −1.31, −7.63]). No significant variation over time was observed for morning SpO_2_ (97 ± 1%, *p* = 0.15) or sleep quality perception (3.8 ± 0.4 a.u., *p* = 0.79). Finally, compared to LHTH4, the temperature was significantly higher in the 12 days following the return to sea level (17.4 ± 2.4 vs. 28.3 ± 3.3°C, *p* < 0.001 [95% CI 13.7, 8.01]) in addition to reduced humidity (68 ± 5.1 vs. 53.3 ± 10.2% RH, *p* = 0.001 [95% CI −6.51, −22.9]).

## 4 DISCUSSION

Our results report that elite racewalkers achieved a modest but significant increase in Hbmass (+1.7 ± 1.9%) after a 4‐week LHTH camp at an altitude of 1720 m. However, 2 weeks after the return to sea level, only RBCV remained elevated compared to the last week at altitude. Finally, PV was significantly increased during heat exposure. Although causation cannot be inferred from our study design, we note that this intervention led to performance success at the subsequent WC events. This suggests that the combination of an increased training load while exposed to low altitude, likely through a training camp effect, followed by tapering in a warm environment, is a valuable competition preparation strategy.

Although an elevation in Hbmass commonly occurs after 4 weeks of LHTH camp to altitudes higher than 2000 m (Gore et al., 2013), findings are considerably more variable at lower altitudes. Indeed, while some LHTH studies have demonstrated improvements in Hbmass (∼4%–5%) after prolonged hypoxic exposures ranging from 1360 to 1800 m (Frese & Friedmann‐Bette, 2010; Sharma et al., 2019; Wachsmuth et al., 2013), other observational results have failed to reveal a significant increase (Gore et al., 1997; Pottgiesser et al., 2009). The modest increase in Hbmass observed in this study (+1.7% for absolute Hbmass and +2.3% for relative Hbmass) (Figure 2) places our findings in line with previous research at altitudes lower than 2000 m, noting that this hypoxic dose could be sufficient in certain, but not all, individuals. Indeed, the large CV% for measurements of both absolute (134%) and relative Hbmass (110%) (Figure 3), and the observation of a positive Hbmass response in most but not all of the cohort (8 out of 10), affirms the typical variability in hematological responses to hypoxic exposure in elite endurance athletes (McLean et al., 2013; Nummela et al., 2021) while there is no evident consensus regarding the influence of sex on Hbmass increase (Raberin et al., 2023).

Nonetheless, our results indicate a marked disparity from the elevation in Hbmass projected from theoretical estimates (1106 km h ≃ 3.6%) (Garvican‐Lewis et al., 2016), outlining the need to consider several factors beyond a given hypoxic dose in the assessment of individual responses to hypoxia. Overall, the consideration of the actual physiological stress (resulting in a decrease in SpO_2_ and eventually a shift in the hemoglobin‐dissociation curve) to a given hypoxic stimulus (e.g., P_I_O_2_) is required to better quantify the actual hypoxic dose (Soo et al., 2020). An evaluation of the hypoxic dose based on « saturation hours » as suggested by Millet et al. (2016) would likely align more closely with the physiological stress incurred during low altitude exposure. The latter, however, requires continuous monitoring of the SpO_2_ which is impractical to record in athletes. Hence, the modest arterial desaturation recorded only at wake‐up in the morning (avg. 97 ± 1%) may not reflect the transitory larger desaturation levels that may occur during training in athletes. Moreover, considering the large intra‐ and inter‐individual variability in the altitude‐induced erythropoietic response (Chapman et al., 1998), such an approach would provide a more detailed understanding of individual responses to an applied hypoxic dose (Soo et al., 2020).

Nevertheless, most of the hematological advantages (e.g., increased Hbmass) observed at the end of the prolonged hypoxic exposure (LHTH4) were virtually entirely dissipated within 14 days following the return to sea level (POST2). This aligns with the existing literature, which consistently reports a swift loss of Hbmass gains within 2 weeks subsequent to a hypoxic dose (Ploszczyca et al., 2018). A neocytolysis phenomenon (i.e., selective destruction of excess RBC produced following stress‐erythropoiesis in hypoxia) has often been mentioned to explain this rapid loss (Alfrey et al., 1997; Rice & Alfrey, 2005). However, Klein and colleagues have recently challenged this hypothesis (Klein et al., 2021), instead suggesting a rapid reduction in RBC production rather than accelerated destruction (Siebenmann et al., 2023). Our data, showing a decrease in reticulocytes (Ret% and Ret#) at POST2 (Figure 4), support this assumption.

A large elevation in PV was recorded at POST2 compared to LHTH4. Since there is no systematic evidence of a PV rebound following return to sea level after altitude (Siebenmann et al., 2015, 2023), our findings likely reflect the response to the cumulative effects of tapering in conjunction with a higher environmental temperature. Indeed, increased training loads (as observed in our study) may lead to discernible changes/increases in BV (Astolfi et al., 2021), and more particularly in PV. The decrease in [Hb] and Hct usually observed during a tapering phase in endurance‐trained subjects suggests that a hemodilution occurs (Mujika et al., 2000). Secondly, we note that athletes were exposed to drastic temperature changes: from a mild temperature during LHTH4 (17.4 ± 2.4°C; 68 ± 5.1% RH) to a considerably higher temperature in the 12 days following the return to sea level (28.3 ± 3.3°C; 53.3 ± 10.2% RH). Exposure to a warmer environment may explain the increase in actual PV, since it is a principal feature of heat acclimatization (Garrett et al., 2014). Therefore, given the extensive evidence of enhanced endurance performance in hot conditions following heat acclimatization (Pandolf et al., 1977; Racinais et al., 2015), and noting that temperatures during the WC races were very similar to those encountered during the tapering phase, it is probable that final performances were underpinned by the increased PV and its enhancement of muscle perfusion, stroke volume, and the related maximum cardiac output (Fellmann, 1992).

Furthermore, cross‐acclimation strategies combining hypoxia and heat exposures have recently been suggested (Gibson et al., 2017; Sotiridis et al., 2022). Indeed, improved maximal oxygen uptake in hot conditions (35°C; 50% RH) was observed following 10 days of continuous hypoxic acclimatization combined with moderate‐intensity exercise training (Sotiridis et al., 2018). Therefore, considering the warm conditions reported during the competition (30°C; 44% RH), a beneficial effect of LHTH on performance in the heat is possible. Moreover, while both conditions simultaneously may not be advisable (McCleave et al., 2017, 2019; Sotiridis et al., 2019), sequential periodization could maximize adaptations (Girard et al., 2024; Mujika et al., 2019; Rendell et al., 2017). Although the literature is scarce, a recent case study showed promising results by periodizing altitude training and heat acclimation before major international competitions in a world‐class level racewalker (Carr et al., 2020). Hence, although the present study design does not allow us to determine how these two environmental stimuli interact, these results support further investigations successively integrating altitude and heat exposure, particularly with more severe altitudes inducing reduced SpO_2_.

Beyond possible hematological improvements, altitude training camps in a natural environment provide athletes with the opportunity to focus exclusively on training and rest (Bonetti & Hopkins, 2009), resulting in higher overall training loads. Accordingly, a large elevation in weekly training distance (+28%), duration (+30%), and load (+71%) was reported during the intervention. This is in agreement with reports from numerous LHTH studies in which an increased training load was observed during the altitude phase (Bonne et al., 2014; Pugliese et al., 2014; Sharma et al., 2018, 2019; Solli et al., 2017). Therefore, a training camp effect most likely occurred during the altitude exposure and certainly explains a substantial part of the hematological benefits observed after 4 weeks of LHTH. Engaging in high‐intensity training prior to the taper period is key to maximizing physiological and performance adaptations (Aubry et al., 2014; Mujika, 2010). However, higher altitudes may be associated with detrimental effects on hypoxia‐altered recovery, such as compromised sleep quality (Bloch et al., 2015; Roberts et al., 2019).

Therefore, low altitude (<2000 m) LHTH strategies may serve as a straightforward and effective approach to maintain absolute exercise intensity and increase training load toward competitive phases whilst achieving a modest effect on Hbmass (Pugliese et al., 2014; Sharma, 2022). Indeed, since the combination of hypoxic dose and training load changes experienced in real‐life altitude cannot be individually discriminated, a positive effect of an increased training load on Hbmass cannot be excluded. For example, subsequent to PV adaptation, an acceleration of erythropoiesis is generally observed following endurance training (Montero et al., 2017). Therefore, although elevating training load within a brief timeframe does not seem to be sufficient for inducing substantial variations in Hbmass (Astolfi et al., 2021; Bejder et al., 2017), a synergistic impact of both hypoxic dose and increased training load may be hypothesized.

### 4.1 Strengths and limitations of the study

The principal merit of the present investigation lies in its alignment with the lead‐up to the 2023 WC, offering a rare perspective on collecting physiological measures in ecologically valid hypoxic interventions with elite endurance athletes during their preparation for a major competition. Nevertheless, some clear limitations need to be mentioned. Primarily, due to geographical constraints, the intended pre‐LHTH baseline measurements could not be executed as initially intended. Although the timeframe required for new RBC to mature makes it unlikely that particular parameters (e.g., Hbmass) were perturbed at the time of the baseline collection timepoint, other markers (e.g., PV) may have been immediately affected by hypoxic exposure.

In addition, the lack of a significantly decreased SpO_2_ challenges the assessment of hypoxic exposure as a contributing parameter to the physiological changes observed in the present study. Nevertheless, hypoxic conditions being defined as a combination of barometric pressure and an inspired fraction of oxygen that results in a P_I_O_2_ lower than a normoxic value of 150 mmHg (Conkin & Wessel, 2008), the conditions at the training camp (PIO2 = 121 mmHg) can legitimately be considered hypoxic. Also, due to the small number of subjects involved and several missing values, statistical results should be carefully interpreted. Finally, despite the inherent complexity of intervening in the preparation of elite endurance athletes for major events, the absence of a control group engaged in equivalent training regimens at sea level remains a major limitation to be mentioned.

## 5 CONCLUSION

Given the low erythropoietic response, we propose that performance increases that occur after prolonged exposure to low altitude should not be expected or explained solely by an increased Hbmass. Our findings suggest that the high‐level performances observed in elite racewalkers may be attributed at least partially to an elevated training load combined with low hypoxic exposure and possible heat acclimatization. Finally, in contributing to the debate around the ergogenic effects of LHTH strategies, our observations of highly successful performances of elite racewalkers following the protocols involved in this study support the relevance of low altitude training when combined with increased training load and subsequent heat exposure in competition preparation phases for elite endurance athletes.

## AUTHOR CONTRIBUTIONS

Bastien Krumm, Raphael Faiss, Brent Vallance & Louise Burke designed the study. Bastien Krumm, Raphael Faiss, Jonas J. Saugy, Johan Garcia, Janne Bouten & Franck Brocherie contributed to data collection. Bastien Krumm drafted the first version of the manuscript, and all authors revised it critically. All authors read and approved the final version of the manuscript.

## CONFLICT OF INTEREST STATEMENT

There are no conflicts of interest to disclose.

## PATIENT CONSENT STATEMENT

All participants provided informed consent.

## Data Availability

Data are available from the corresponding author upon reasonable request.
